# Supplementary figures and images for: HIV-1 envelope glycoprotein modulates CXCR4 clustering and dynamics on the T cell membrane
Source: eLife. 2026 May 12;15:RP110354. doi: 10.7554/eLife.110354 (PMC13167113; doi:10.7554/eLife.110354)

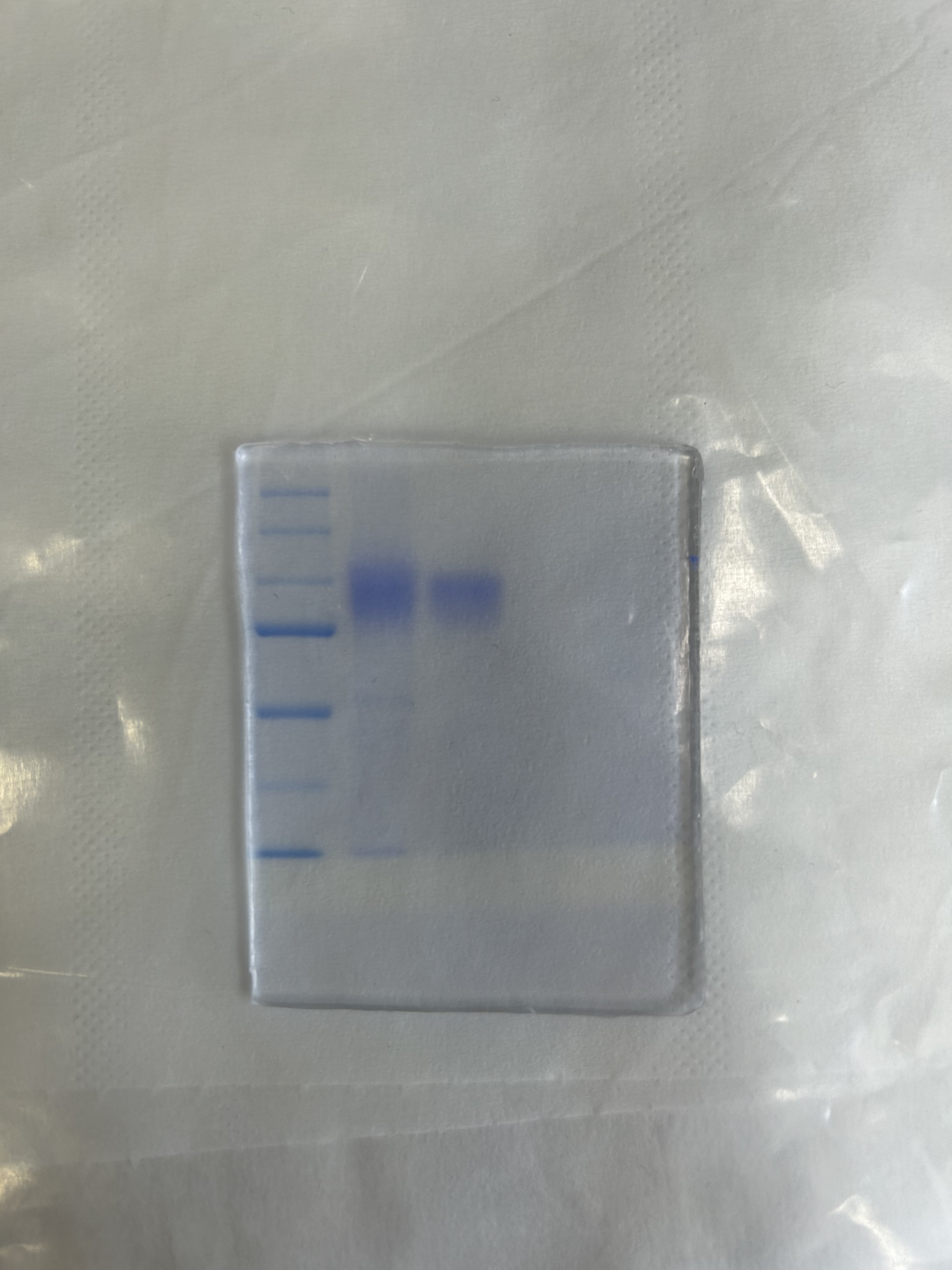

Supplement: Figure 1—figure supplement 1—source data 1. [file elife-110354-fig1-figsupp1-data1.zip › Figure 1-Figure supplement 1-Source data 1/Figure 1-Figure supplement 1-Source data 1.jpg]

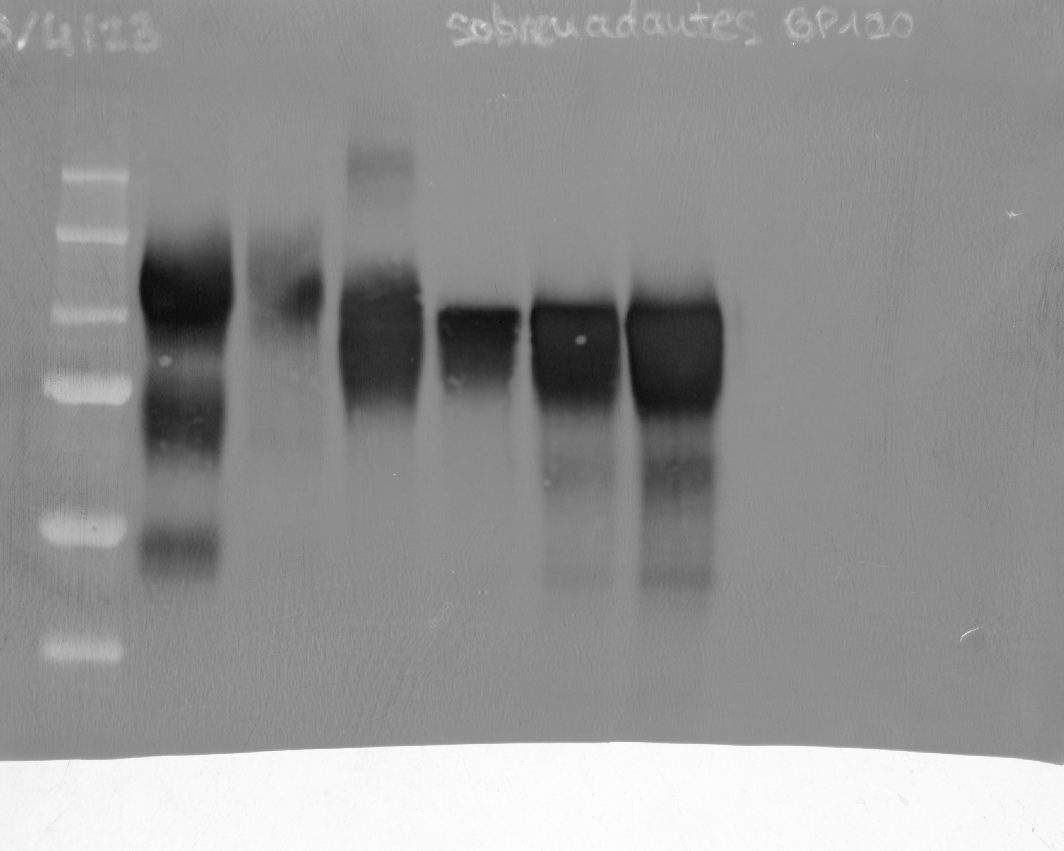

Supplement: Figure 1—figure supplement 1—source data 3. [file elife-110354-fig1-figsupp1-data3.zip › Figure 1-Figure supplement 1-Source data 3/FIG S1 B 416 MM 2023-04-19 11h55m03s(Composite) (1).tif]

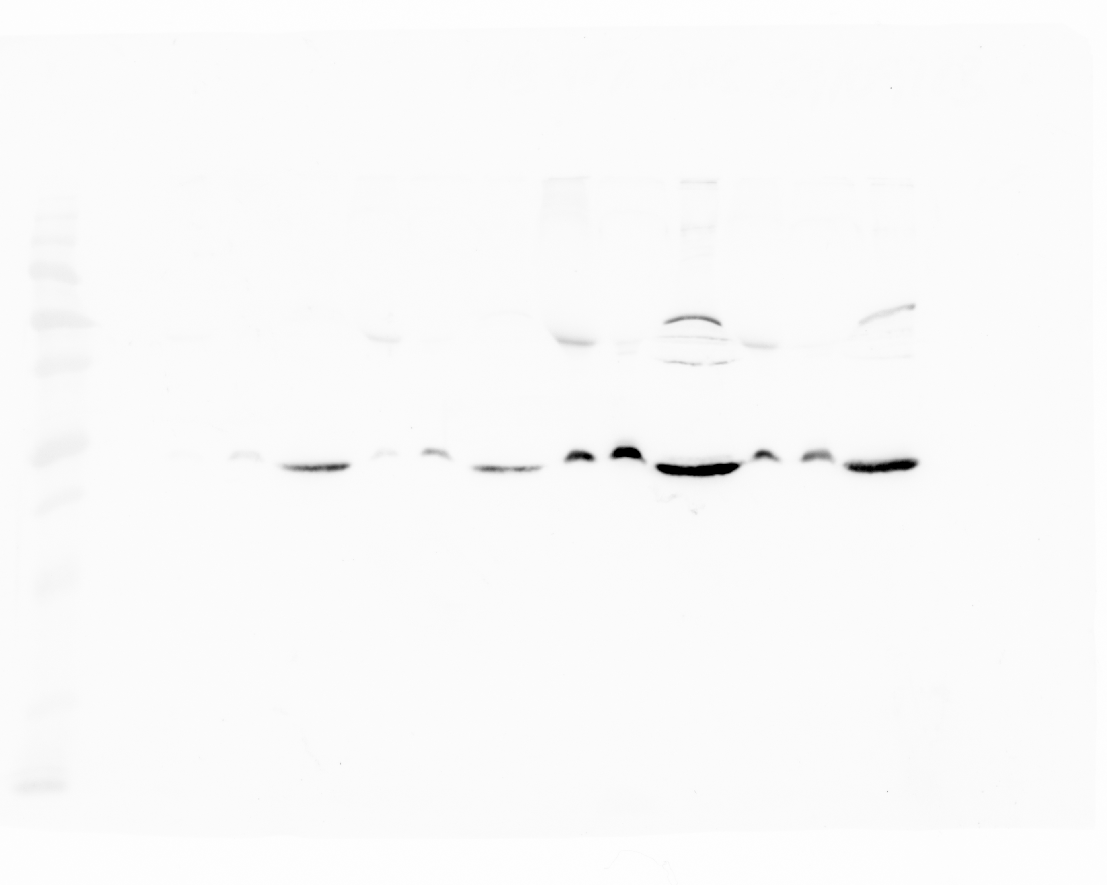

Supplement: Figure 1—figure supplement 2—source data 1. [file elife-110354-fig1-figsupp2-data1.zip › Figure 2-Figure supplement 1-Source data 1/416 MM 2023-10-02 14h47m03s.tif]

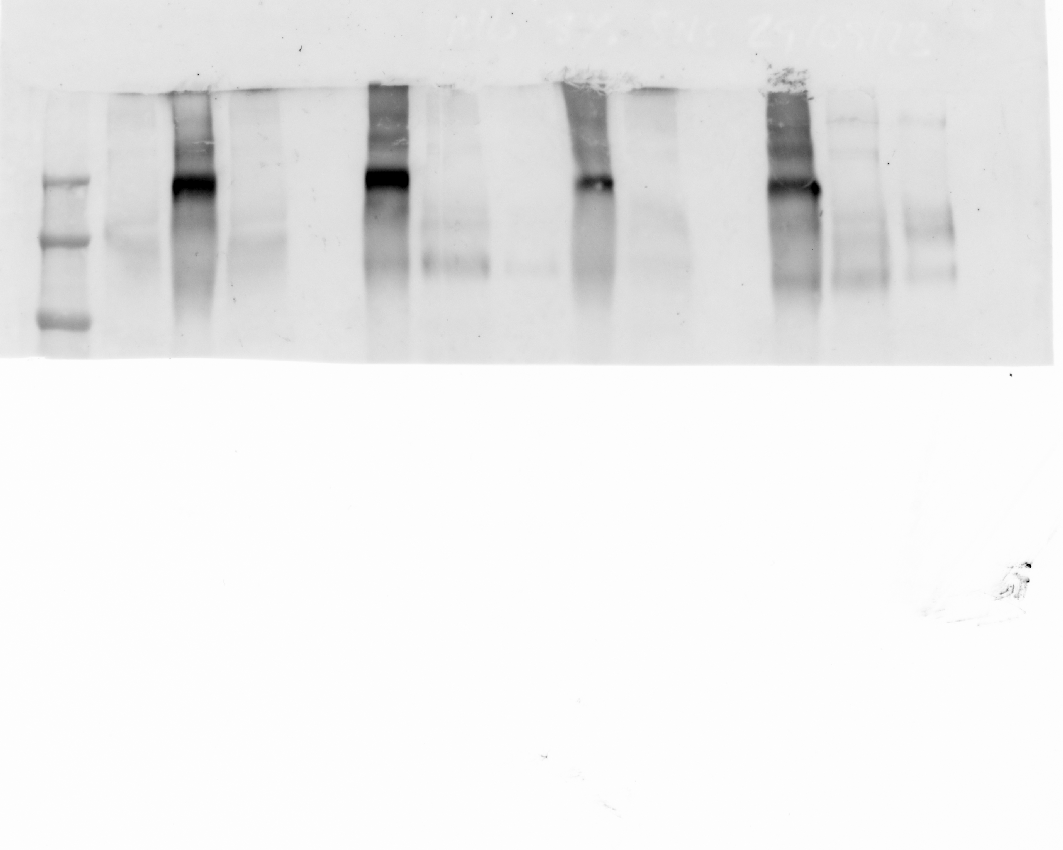

Supplement: Figure 1—figure supplement 2—source data 1. [file elife-110354-fig1-figsupp2-data1.zip › Figure 2-Figure supplement 1-Source data 1/416 MM 2023-10-02 14h44m01s.tif]

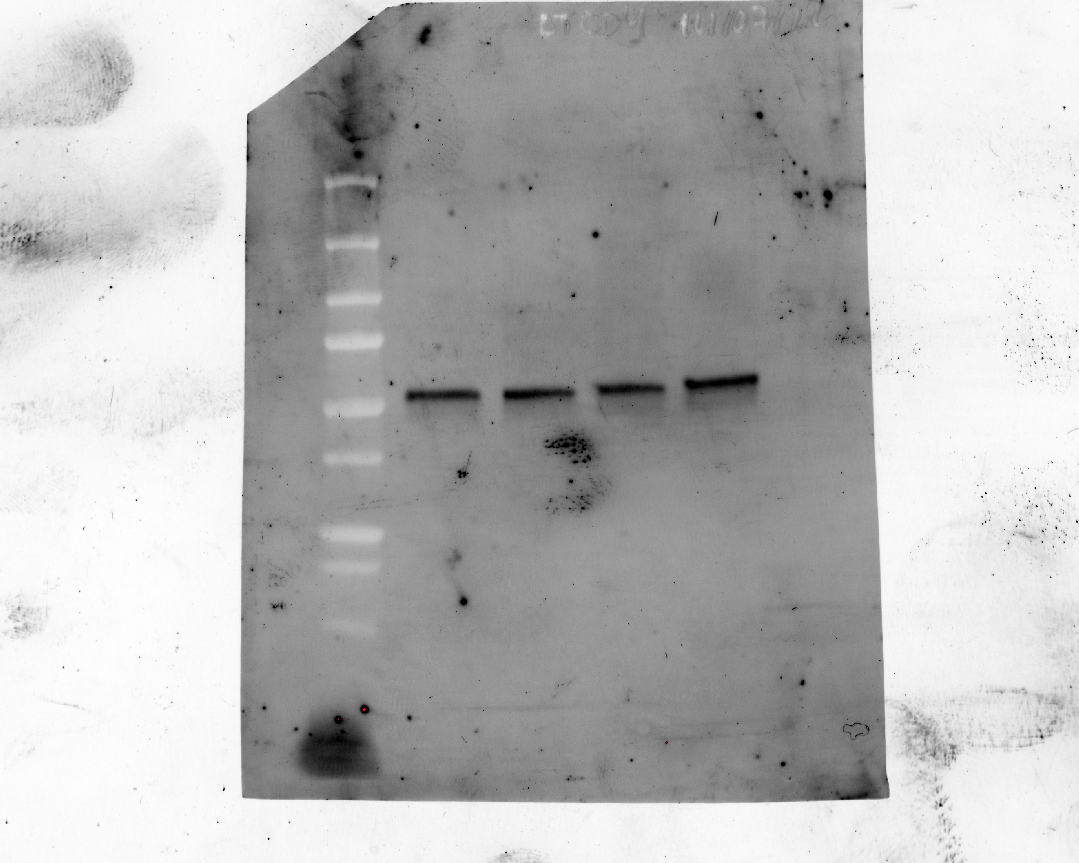

Supplement: Figure 1—figure supplement 2—source data 3. [file elife-110354-fig1-figsupp2-data3.zip › Figure 1-Figure supplement 2-Source data 3/416 MM 2022-07-19 16h08m23s.tif]

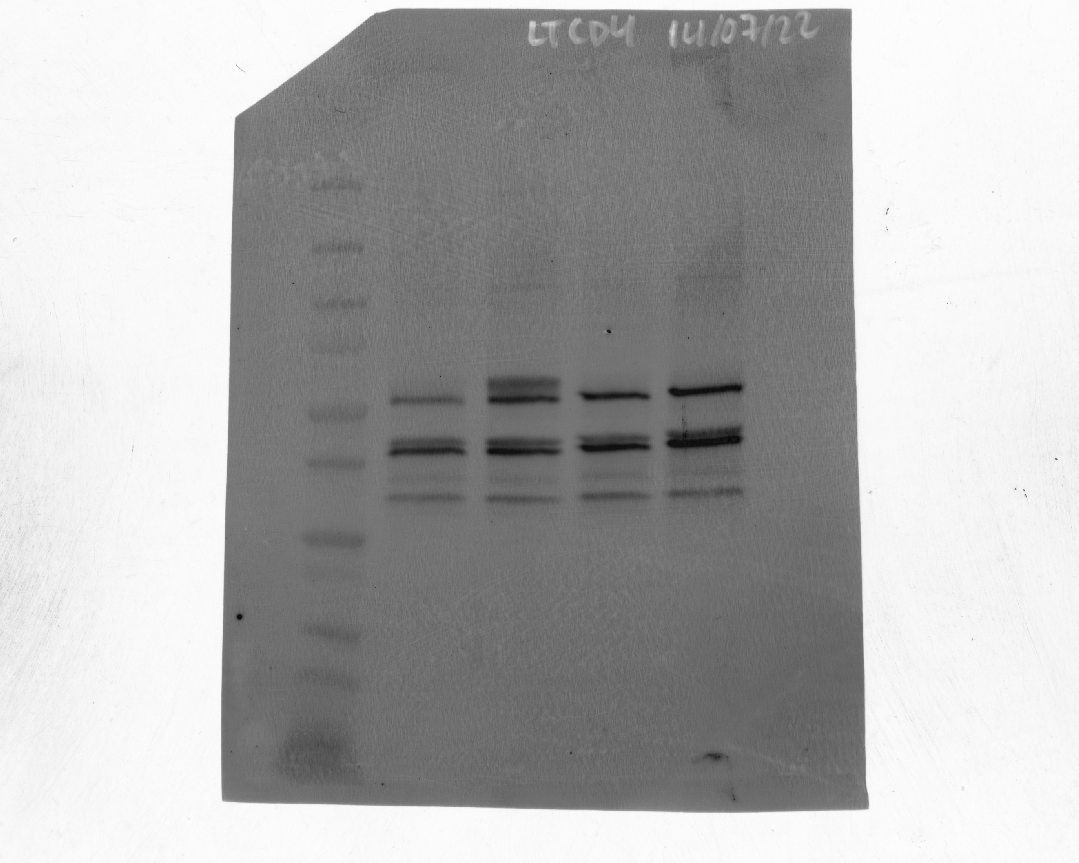

Supplement: Figure 1—figure supplement 2—source data 3. [file elife-110354-fig1-figsupp2-data3.zip › Figure 1-Figure supplement 2-Source data 3/416 MM 2022-07-18 13h21m06s.tif]

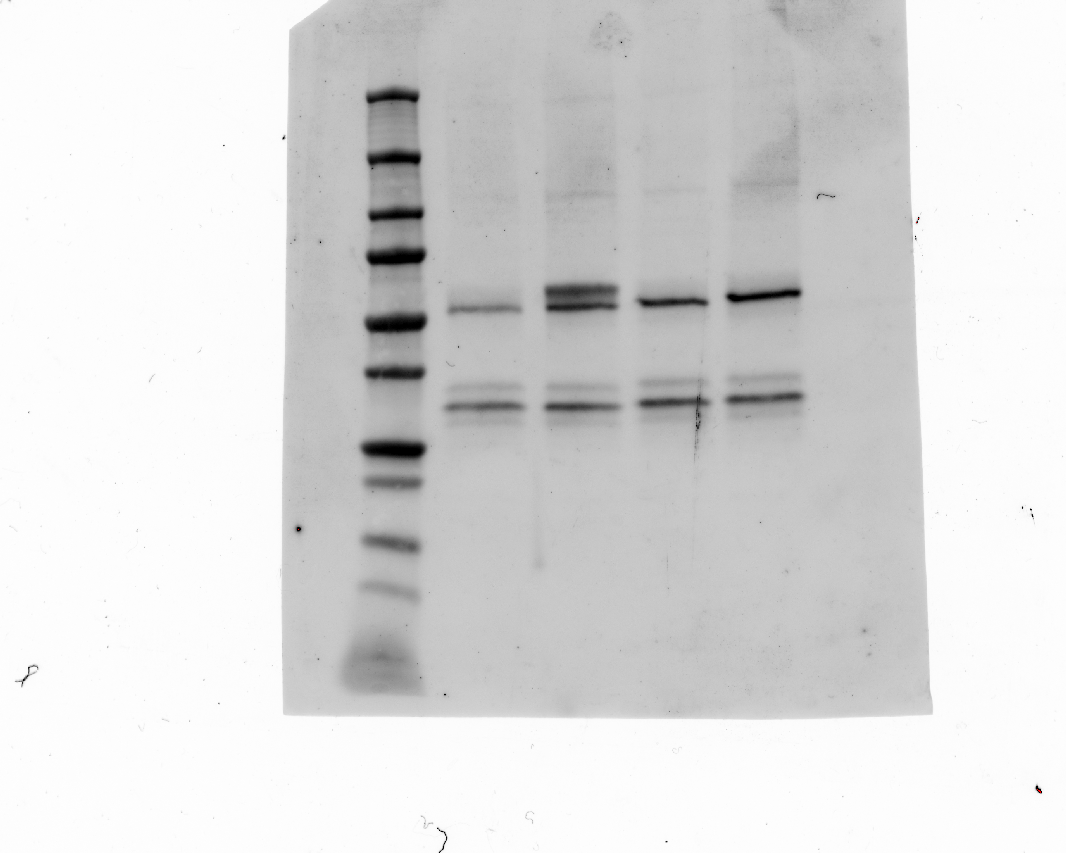

Supplement: Figure 1—figure supplement 2—source data 3. [file elife-110354-fig1-figsupp2-data3.zip › Figure 1-Figure supplement 2-Source data 3/pLCK Adri 15.07.22.tif]
