# Supplementary material for: HIV-1 envelope glycoprotein modulates CXCR4 clustering and dynamics on the T cell membrane
Source: eLife. 2026 May 12;15:RP110354. doi: 10.7554/eLife.110354 (PMC13167113; doi:10.7554/eLife.110354)
Supplement: Figure 1—figure supplement 1—source data 2. — Generation of functional recombinant X4-gp120. Coomassie blue-stained polyacrylamide gel of 5 μg of purified recombinant X4-gp120 and commercial gp120 (gp120), used as a control. Original files for Coomassie blue-stained polyacrylamide gel displayed in Figure 1—figure supplement 1—source data 1. [file elife-110354-fig1-figsupp1-data2.zip › Figure 1-Figure supplement 1-Source data 2.pdf]

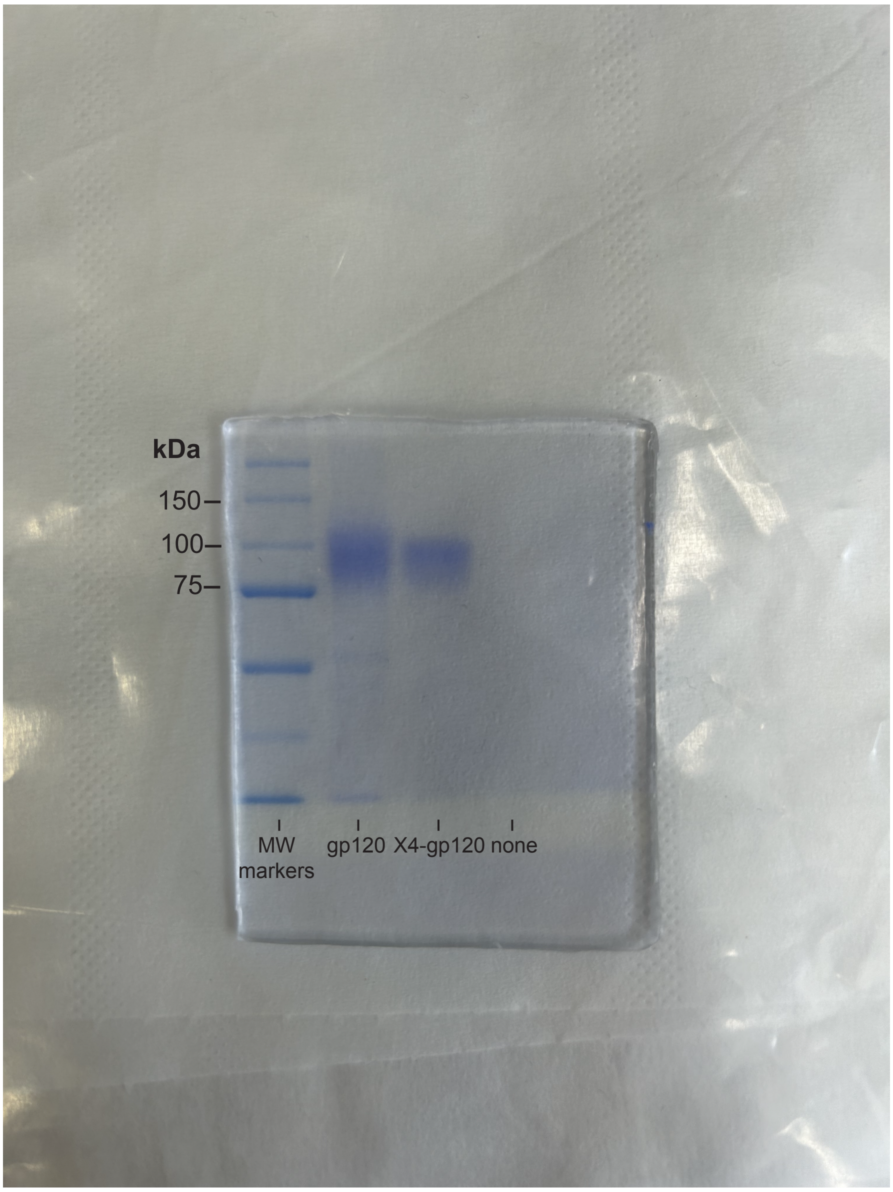

Figure 1-Figure supplement 1-Source data 2

PDF file containing original Coomassie blue-stained polyacrylamide gel for Figure 1-Figure supplement 1A.

Generation of functional recombinant X4-gp120. Coomassie blue-stained polyacrylamide gel of 5 µg of purified recombinant X4-gp120 and commercial gp120 (gp120), used as a control.

Original files for Coomassie blue-stained polyacrylamide gel displayed in Figure 1-Figure supplement 1-Source data 1.
