# Supplementary material for: HIV-1 envelope glycoprotein modulates CXCR4 clustering and dynamics on the T cell membrane
Source: eLife. 2026 May 12;15:RP110354. doi: 10.7554/eLife.110354 (PMC13167113; doi:10.7554/eLife.110354)
Supplement: Figure 1—figure supplement 1—source data 4. — Original membrane corresponding to Figure 1—figure supplement 1, panel B. Western blot of different amounts (μg indicated) of different batches of recombinant X4-gp120 and commercial gp120 (5 μg), used as control, analyzed with an anti-gp120 mAb. Batch#3 was selected for further assays. Original files for western blot analysis displayed in Figure 1—figure supplement 1—source data 3. [file elife-110354-fig1-figsupp1-data4.zip › Figure 1-Figure supplement 1-Source data 4.pdf]

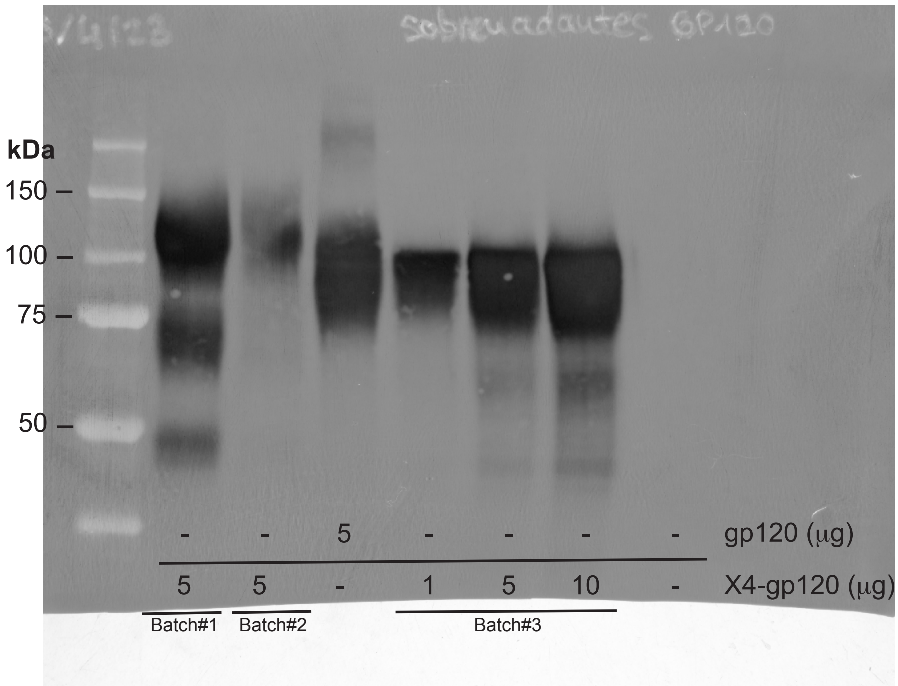

Figure 1-Figure supplement 1-Source data 4.

PDF file containing original western blot for Figure 1-Figure supplement 1B.

Original membrane corresponding to Figure 1-Figure supplement 1, panel B. Western blot of different amounts (μg indicated) of different batches of recombinant X4-gp120 and commercial gp120 (5 μg), used as control, analyzed with an anti-gp120 mAb. Batch#3 was selected for further assays.

Original files for western blot analysis displayed in Figure 1-Figure supplement 1-Source data 3.
