# Supplementary material for: HIV-1 envelope glycoprotein modulates CXCR4 clustering and dynamics on the T cell membrane
Source: eLife. 2026 May 12;15:RP110354. doi: 10.7554/eLife.110354 (PMC13167113; doi:10.7554/eLife.110354)
Supplement: Figure 1—figure supplement 2—source data 2. — Original membrane corresponding to Figure 1—figure supplement 2, panel A. Western blot of lysed Jurkat cells activated with CXCL12 (50 nM) or X4-gp120 Batch#3 (0.05; 0.1; 0.3 μg/ml) at the indicated time points and analyzed with anti-pAkt and pERK1/2 antibodies. Membranes were reblotted with an anti-tubulin mAb as a loading control. Molecular markers (kDa) are also shown. Figure 1—figure supplement 2, panel A shows western blot of lysed Jurkat cells activated with CXCL12 (50 nM) and X4-gp120 Batch#3 0.3 μg/ml. Original files for western blot analysis displayed in Figure 1—figure supplement 2—source data 1. [file elife-110354-fig1-figsupp2-data2.zip › Figure 1-Figure supplement 2-Source data 2.pdf]

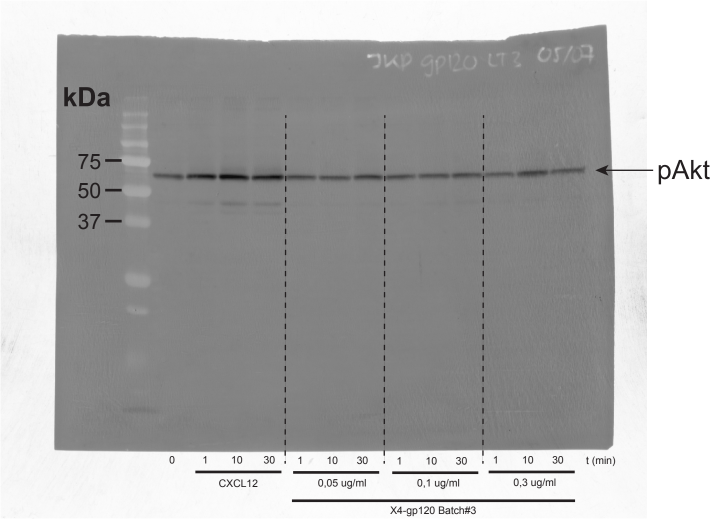

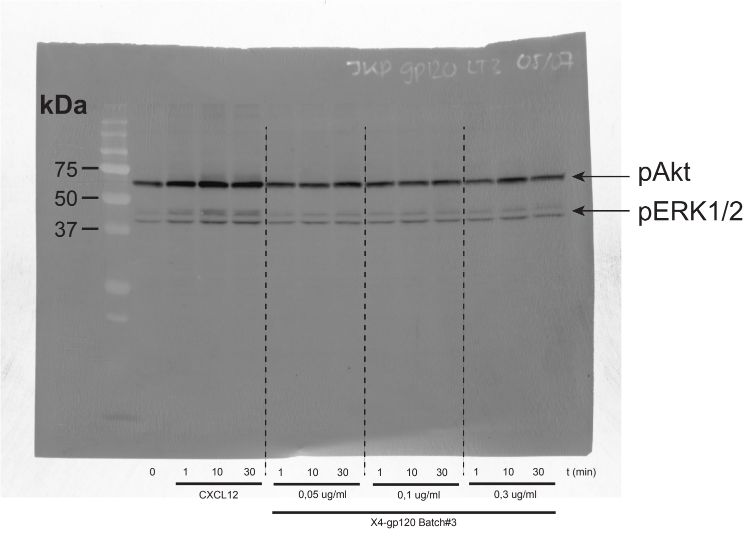

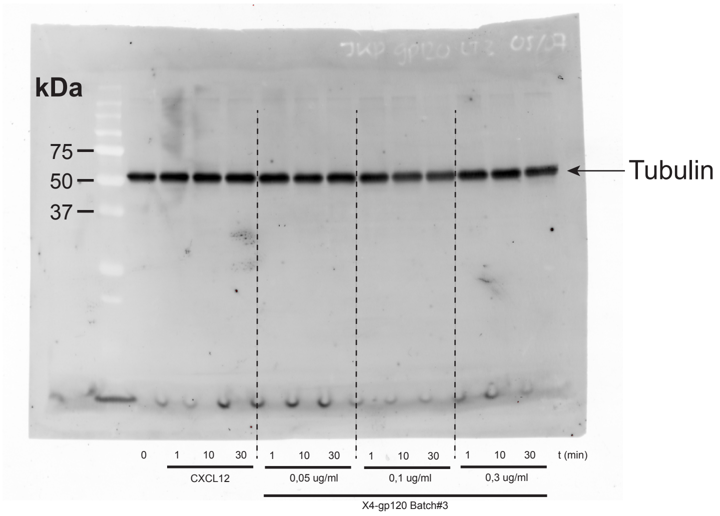

Figure 1-Figure supplement 2-Source data 2.

PDF file containing original western blot for Figure 1-Figure supplement 2A.

Original membrane corresponding to Figure 1-Figure supplement 2, panel A. Western blot of lysed Jurkat cells activated with CXCL12 (50 nM) or X4-gp120 Batch#3 (0.05; 0.1; 0.3 ug/ml) at the indicated time points and analyzed with anti-pAkt and pERK1/2 antibodies. Membrane were reblotted with an anti-tubulin mAb as a loading control. Molecular markers (kDa) are also shown. Figure 1-Figure supplement 2, panel A shows western blot of lysed Jurkat cells activated with CXCL12 (50 nM) and X4-gp120 Batch#3 0.3 ug/ml.

Original files for western blot analysis displayed in Figure 1-Figure supplement 2-Source data 1.
