# Supplementary material for: HIV-1 envelope glycoprotein modulates CXCR4 clustering and dynamics on the T cell membrane
Source: eLife. 2026 May 12;15:RP110354. doi: 10.7554/eLife.110354 (PMC13167113; doi:10.7554/eLife.110354)
Supplement: Figure 1—figure supplement 2—source data 4. — Original membrane corresponding to Figure 1—figure supplement 2, panel B. Western blot of primary CD4+ T blast lysates activated with CXCL12 (50 nM) and X4-gp120 Batch#3 (0.3 μg/ml) at the indicated time points and analyzed using anti-pLck and pERK1/2 antibodies. Membranes were reblotted with an anti-tubulin mAb as a loading control. Molecular markers (kDa) are also shown. Figure 1—figure supplement 2, panel B shows western blot of lysed Jurkat cells activated with X4-gp120 Batch#3 0.3 μg/ml. Original files for western blot analysis displayed in Figure 1—figure supplement 2—source data 3. [file elife-110354-fig1-figsupp2-data4.zip › Figure 1-Figure supplement 2-Source data 4.pdf]

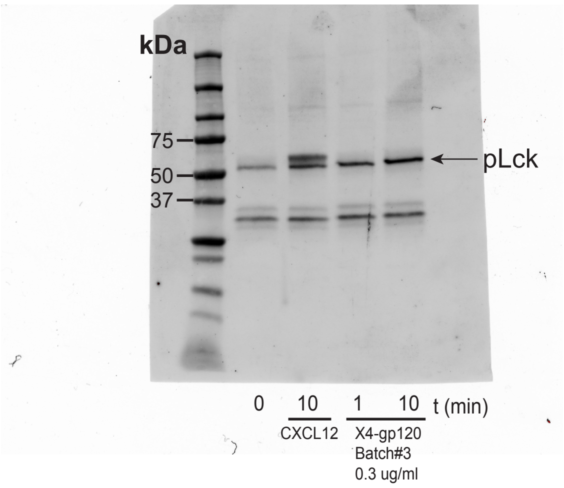

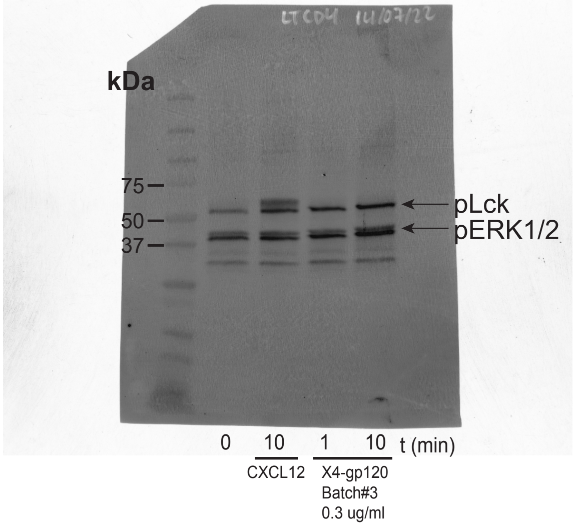

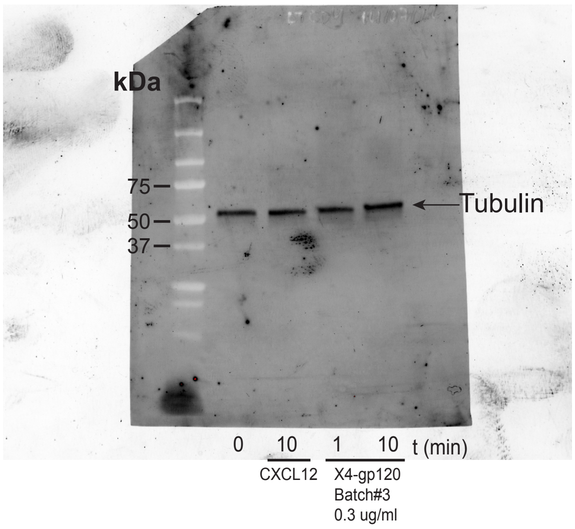

Figure 1-Figure supplement 2-Source data 4.

PDF file containing original western blot for Figure 1-Figure supplement 2B.

Original membrane corresponding to Figure 1-Figure supplement 2, panel B. Western blot of primary $CD4^+$ T blast lysates activated with CXCL12 (50nM) and X4-gp120 Batch#3 (0.3 ug/ml) at the indicated time points and analyzed using anti-pLck and -pERK1/2 antibodies. Membrane were reblotted with an anti-tubulin mAb as a loading control. Molecular markers (kDa) are also shown. Figure 1-Figure supplement 2, panel B shows western blot of lysed Jurkat cells activated with X4-gp120 Batch#3 0.3 ug/ml.

Original files for western blot analysis displayed in Figure 1-Figure supplement 2-Source data 3.
