# Supplementary material for: HIV-1 envelope glycoprotein modulates CXCR4 clustering and dynamics on the T cell membrane
Source: eLife. 2026 May 12;15:RP110354. doi: 10.7554/eLife.110354 (PMC13167113; doi:10.7554/eLife.110354)
Supplement: Figure 2—figure supplement 1—source data 2. — Original membranes corresponding to Figure 2—figure supplement 1, panel B. Culture media of transfected HEK-293T cells (cm) with different constructs, as indicated, and the corresponding clarified samples (cs) containing the VLPs or the LVPs generated, were analyzed by western blot with anti-p24, and -gp120 mAbs. The anti-p24 mAb also recognizes Pr55Gag, a precursor of p24 presents in immature particles. Molecular weight markers are indicated (kDa). Figure 2—figure supplement 1, panel B shows the last six lanes of these membranes reorganized to separate LVPs from VLPs. Original files for western blot analysis displayed in Figure 2—figure supplement 1—source data 1. [file elife-110354-fig2-figsupp1-data2.zip › Figure 2-Figure supplement 1-Source data 2.pdf]

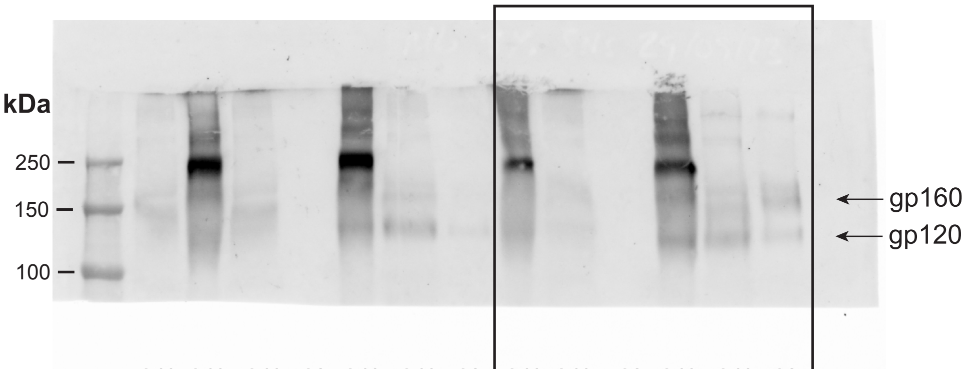

	cm	cm	cm	cs	cm	cm	cs	cm	cm	cs	cm	cm	cs
pHXB2env	-	-	-	-	10	10	10	-	-	-	10	10	10
psPAX2	-	-	-	-	-	-	-	7	7	7	7	7	7
pRRE	-	10	10	10	10	10	10	-	-	-	-	-	-
Rev	-	2	2	2	2	2	2	-	-	-	-	-	-
plentiGFP	-	8	-	-	8	-	-	8	-	-	8	-	-

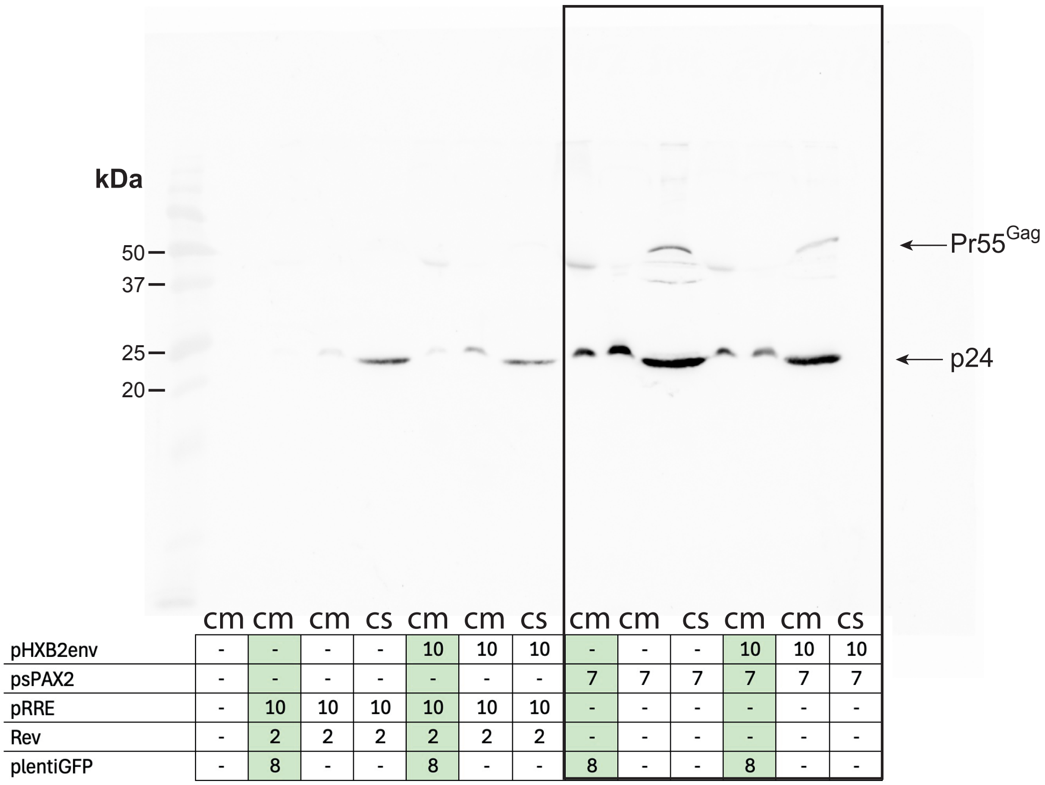

	cm	cm	cm	cs	cm	cm	cs	cm	cm	cs	cm	cm	cs
pHXB2env	-	-	-	-	10	10	10	-	-	-	10	10	10
psPAX2	-	-	-	-	-	-	-	7	7	7	7	7	7
pRRE	-	10	10	10	10	10	10	-	-	-	-	-	-
Rev	-	2	2	2	2	2	2	-	-	-	-	-	-
plentiGFP	-	8	-	-	8	-	-	8	-	-	8	-	-

cm (culture media)
cs (clarified simples dil 1/25)

LVPs VLPs

Figure 2-Figure supplement 1-Source data 2.

PDF file containing original western blots for Figure 2-Figure supplement 1B.

Original membranes corresponding to Figure 2-Figure supplement 1, panel B. Culture media of transfected HEK-293T cells (cm) with different constructs, as indicated, and the corresponding clarified samples (cs) containing the VLPs or the LVPs generated, were analyzed by western blot with anti-p24, and -gp120 mAbs. The anti-p24 mAb also recognizes Pr55^{Gag}, a precursor of p24 presents in immature particles. Molecular weight markers are indicated (kDa). Figure 2-Figure supplement 1, panel B shows the last six lanes of these membranes reorganized to separate LVPs from VLPs.

Original files for western blot analysis displayed in Figure 2-Figure supplement 1-Source data 1.
